# Poly[(μ_5_-2,6-dimethyl­pyridine-3,5-dicarboxyl­ato)zinc]

**DOI:** 10.1107/S1600536811024172

**Published:** 2011-06-25

**Authors:** Ming-Xing Zhang, Xin Chen, Yi Zhu

**Affiliations:** a College of Chemistry, Chongqing Normal University, Chongqing 400047, People’s Republic of China; b College of Life Science, Chongqing Normal University, Chongqing 400047, People’s Republic of China

## Abstract

In the polymeric title complex, [Zn(C_9_H_7_NO_4_)]_*n*_, the Zn^II^ cation is located on a twofold rotation axis and is coordinated by five 2,6-dimethyl­pyridine-3,5-dicarboxyl­ate (mpdc) anions in a distorted ZnNO_4_ trigonal–bipyramidal geometry. The mpdc anion is also located on the twofold rotation axis and bridges five Zn^II^ cations, forming the three-dimensional polymeric complex. Weak C—H⋯π inter­actions are present in the crystal structure.

## Related literature

For a related structure, see: Huang *et al.* (2007 ▶). For background to metal-organic frameworks (MOFs), see: Long & Yaghi (2009 ▶); Zhao *et al.* (2003 ▶).
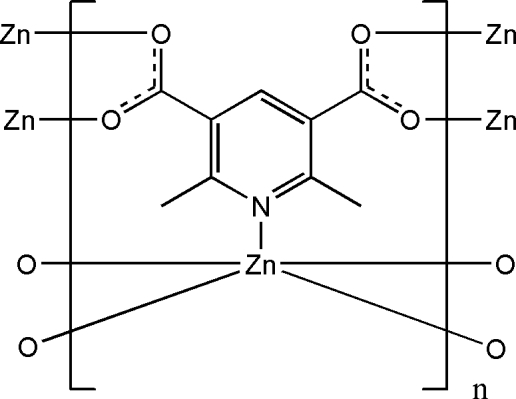


## Experimental

### 

#### Crystal data

[Zn(C_9_H_7_NO_4_)]
*M* *_r_* = 258.53
Monoclinic, 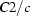

*a* = 8.578 (7) Å
*b* = 14.016 (11) Å
*c* = 7.382 (7) Å
β = 112.176 (17)°
*V* = 821.9 (12) Å^3^
*Z* = 4
Mo *K*α radiation
μ = 2.98 mm^−1^
*T* = 293 K
0.30 × 0.25 × 0.16 mm

#### Data collection

Rigaku Mercury2 diffractometer
Absorption correction: multi-scan (*CrystalClear*; Rigaku, 2005 ▶) *T*_min_ = 0.469, *T*_max_ = 0.647
2615 measured reflections
732 independent reflections
709 reflections with *I* > 2σ(*I*)
*R*_int_ = 0.022

#### Refinement

*R*[*F* ^2^ > 2σ(*F* ^2^)] = 0.023
*wR*(*F* ^2^) = 0.067
*S* = 1.00
732 reflections
71 parameters
1 restraint
H-atom parameters constrained
Δρ_max_ = 0.50 e Å^−3^
Δρ_min_ = −0.59 e Å^−3^

### 

Data collection: *CrystalClear* (Rigaku, 2005 ▶); cell refinement: *CrystalClear*; data reduction: *CrystalClear*; program(s) used to solve structure: *SHELXS97* (Sheldrick, 2008 ▶); program(s) used to refine structure: *SHELXL97* (Sheldrick, 2008 ▶); molecular graphics: *DIAMOND* (Brandenburg, 2008 ▶) and *ORTEP-3* (Farrugia, 1997 ▶); software used to prepare material for publication: *PLATON* (Spek, 2009 ▶).

## Supplementary Material

Crystal structure: contains datablock(s) I, global. DOI: 10.1107/S1600536811024172/xu5247sup1.cif

Structure factors: contains datablock(s) I. DOI: 10.1107/S1600536811024172/xu5247Isup2.hkl

Additional supplementary materials:  crystallographic information; 3D view; checkCIF report

## Figures and Tables

**Table 1:** Selected bond lengths (Å)

Zn1—O1	2.207 (3)
Zn1—O2^i^	1.977 (2)
Zn1—N1^ii^	2.089 (3)

Symmetry codes: (i) 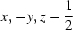; (ii) 
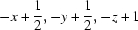
.

**Table 2:** Hydrogen-bond geometry (Å, °) *Cg* is the centroid of the pyridine ring.

*D*—H⋯*A*	*D*—H	H⋯*A*	*D*⋯*A*	*D*—H⋯*A*
C5—H5*C*⋯*Cg*^ii^	0.96	2.67	3.573 (4)	158

Symmetry code: (ii) 
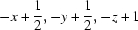
.

**Fig. 1.**
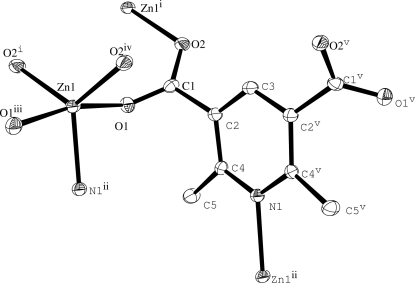
The coordination environments of Zinc ions, showing 30% probability displacement ellipsoids and hydrogen atoms have been removed for clarity. Symmetry codes: (i) -x, -y, -z+1; (ii) -x + 1/2, -y + 1/2,-z + 1; (iii) -x, y, -z+1/2; (iv) x, -y, z - 1/2; (v) -x + 1, +y, -z + 3/2.

**Fig. 2.**
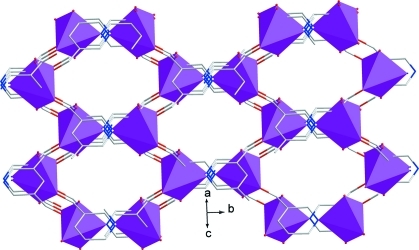
The presentation of the 3-D zeolite-like architecture. Methyl groups and hydrogen atoms have been removed for clarity. Polyhedra represent the ZnNO4 groups.

## supplementary crystallographic information

### Comment

Recently, research on metal-organic frameworks (MOFs) has become of increasing interest (Long & Yaghi, 2009). However, it is still a great challenge to assemble a predicted structure because there are numerous influences that can play decisive roles on the structure and crystal packing. Fortunately, these uncertainties can be reduced by the use of well selected spacers that have the ability to aggregate metal ions into different secondary building units (Zhao *et al.*, 2003). Herein we reports an interesting five-connected zeolite-like coordination polymer based on highly-substituted pyridinedicarboxylates.

The title compound is a three-dimensional framework built from Zn cations that are linked by mpdc anions. From this arrangement cavities are formed. Zn1 is coordinated by four oxygen atoms from four different CO_2_^-^ groups of mpdc ligands and one pyridyl nitrogen atom from another mpdc ligand. The mpdc ligand bridges five different Zn atoms and favors the construction of the structure with zeolite-like topology.The topology of the title compound is identical with the reported [Cd(mpdc)]_n_ (Huang *et al.*, 2007), but the coordination sphere of cation, the binding mode of the carboxylate group and the synthesis condition are different.

The combination of the dramatic twists between two carboxylate groups in mpdc ligands results in the formation of the intersecting double-stranded helical chain comprised of [Zn(CO_2_)_2_]_n_ (Zn atoms as nodes).

### Experimental

All chemicals were of reagent grade and used as purchased without further purification. A mixture of Zn(NO_3_)_2_.6H_2_O (450 mg, 1.5 mmol), H_2_mpdc (97.5 mg, 0.5 mmol), (Et)_3_N 0.07 mL and H_2_O 10 mL was sealed in a 25 ml stainless steel reactor with Teflon liner and directly heated to 180 °C for 3 days, and then cooled to room temperature. The crystal samples were washed with methanol to give the title compound in about 35% yield (based on H_2_mpdc ligand).

### Refinement

Constraint instruction 'delu 0.001 Zn1 O1' was used in the refinement. All H atoms were placed in geometrically idealized positions (C—H = 0.93 Å) and treated as riding on their parent atoms, with U_iso_(H) = 1.5U_eq_(C) for methyl H atoms and 1.2U_eq_(C) for aromatic H atom.

### Crystal data

**Table:**

[Zn(C_9_H_7_NO_4_)]	*F*(000) = 520
*M**_r_* = 258.53	*D*_x_ = 2.089 Mg m^−^^3^
Monoclinic, *C*2/*c*	Mo *K*α radiation, λ = 0.71073 Å
Hall symbol: -C 2yc	Cell parameters from 535 reflections
*a* = 8.578 (7) Å	θ = 2.9–27.5°
*b* = 14.016 (11) Å	µ = 2.98 mm^−^^1^
*c* = 7.382 (7) Å	*T* = 293 K
β = 112.176 (17)°	Prism, colorless
*V* = 821.9 (12) Å^3^	0.30 × 0.25 × 0.16 mm
*Z* = 4	

### Data collection

**Table:**

Rigaku Mercury2 diffractometer	732 independent reflections
Radiation source: fine-focus sealed tube	709 reflections with *I* > 2σ(*I*)
graphite	*R*_int_ = 0.022
φ and ω scans	θ_max_ = 25.0°, θ_min_ = 2.9°
Absorption correction: multi-scan (*CrystalClear*; Rigaku, 2005)	*h* = −10→10
*T*_min_ = 0.469, *T*_max_ = 0.647	*k* = −14→16
2615 measured reflections	*l* = −8→8

### Refinement

**Table:**

Refinement on *F*^2^	Primary atom site location: structure-invariant direct methods
Least-squares matrix: full	Secondary atom site location: difference Fourier map
*R*[*F*^2^ > 2σ(*F*^2^)] = 0.023	Hydrogen site location: inferred from neighbouring sites
*wR*(*F*^2^) = 0.067	H-atom parameters constrained
*S* = 1.00	*w* = 1/[σ^2^(*F*_o_^2^) + (0.0519*P*)^2^ + 0.6817*P*] where *P* = (*F*_o_^2^ + 2*F*_c_^2^)/3
732 reflections	(Δ/σ)_max_ < 0.001
71 parameters	Δρ_max_ = 0.50 e Å^−^^3^
1 restraint	Δρ_min_ = −0.59 e Å^−^^3^

### Special details

**Table:**

Geometry. All e.s.d.'s (except the e.s.d. in the dihedral angle between two l.s. planes) are estimated using the full covariance matrix. The cell e.s.d.'s are taken into account individually in the estimation of e.s.d.'s in distances, angles and torsion angles; correlations between e.s.d.'s in cell parameters are only used when they are defined by crystal symmetry. An approximate (isotropic) treatment of cell e.s.d.'s is used for estimating e.s.d.'s involving l.s. planes.
Refinement. Refinement of *F*^2^ against ALL reflections. The weighted *R*-factor *wR* and goodness of fit *S* are based on *F*^2^, conventional *R*-factors *R* are based on *F*, with *F* set to zero for negative *F*^2^. The threshold expression of *F*^2^ > σ(*F*^2^) is used only for calculating *R*-factors(gt) *etc*. and is not relevant to the choice of reflections for refinement. *R*-factors based on *F*^2^ are statistically about twice as large as those based on *F*, and *R*- factors based on ALL data will be even larger.

### Fractional atomic coordinates and isotropic or equivalent isotropic displacement parameters (Å^2^)

**Table:**

	*x*	*y*	*z*	*U*_iso_*/*U*_eq_	
Zn1	0.0000	0.08158 (2)	0.2500	0.01616 (18)	
N1	0.5000	0.26938 (18)	0.7500	0.0137 (5)	
O1	0.0625 (2)	0.09690 (11)	0.5672 (2)	0.0189 (4)	
O2	0.20746 (19)	−0.00679 (11)	0.7999 (2)	0.0192 (4)	
C1	0.1949 (3)	0.06763 (15)	0.6979 (3)	0.0147 (5)	
C2	0.3560 (3)	0.12188 (16)	0.7360 (3)	0.0147 (5)	
C3	0.5000	0.0730 (2)	0.7500	0.0168 (7)	
H3	0.5000	0.0066	0.7500	0.020*	
C4	0.3618 (3)	0.22210 (15)	0.7464 (3)	0.0137 (5)	
C5	0.2202 (3)	0.28064 (16)	0.7589 (4)	0.0195 (5)	
H5A	0.2644	0.3278	0.8596	0.029*	
H5B	0.1434	0.2399	0.7893	0.029*	
H5C	0.1621	0.3117	0.6358	0.029*	

### Atomic displacement parameters (Å^2^)

**Table:**

	*U*^11^	*U*^22^	*U*^33^	*U*^12^	*U*^13^	*U*^23^
Zn1	0.0108 (2)	0.0106 (3)	0.0269 (3)	0.000	0.00690 (17)	0.000
N1	0.0128 (13)	0.0115 (13)	0.0166 (12)	0.000	0.0054 (10)	0.000
O1	0.0143 (8)	0.0178 (8)	0.0228 (7)	0.0007 (7)	0.0050 (7)	0.0013 (6)
O2	0.0146 (8)	0.0135 (8)	0.0277 (8)	−0.0012 (6)	0.0061 (6)	0.0048 (6)
C1	0.0143 (12)	0.0124 (11)	0.0202 (11)	−0.0012 (9)	0.0095 (9)	−0.0039 (8)
C2	0.0145 (11)	0.0121 (12)	0.0175 (10)	−0.0004 (9)	0.0058 (9)	0.0007 (8)
C3	0.0161 (17)	0.0107 (16)	0.0226 (17)	0.000	0.0064 (14)	0.000
C4	0.0111 (11)	0.0133 (11)	0.0165 (10)	−0.0011 (8)	0.0049 (8)	−0.0001 (8)
C5	0.0160 (11)	0.0150 (12)	0.0302 (12)	0.0005 (9)	0.0118 (10)	−0.0021 (9)

### Geometric parameters (Å, °)

**Table:**

Zn1—O1	2.207 (3)	O2—Zn1^iii^	1.977 (2)
Zn1—O1^i^	2.207 (3)	C1—C2	1.507 (3)
Zn1—O2^ii^	1.977 (2)	C2—C3	1.382 (3)
Zn1—O2^iii^	1.977 (2)	C2—C4	1.407 (3)
Zn1—N1^iv^	2.089 (3)	C3—C2^v^	1.382 (3)
N1—C4	1.349 (3)	C3—H3	0.9300
N1—C4^v^	1.349 (3)	C4—C5	1.497 (3)
N1—Zn1^iv^	2.089 (3)	C5—H5A	0.9600
O1—C1	1.250 (3)	C5—H5B	0.9600
O2—C1	1.267 (3)	C5—H5C	0.9600
			
O2^iii^—Zn1—O2^ii^	115.94 (11)	O2—C1—C2	116.0 (2)
O2^iii^—Zn1—N1^iv^	122.03 (5)	C3—C2—C4	118.6 (2)
O2^ii^—Zn1—N1^iv^	122.03 (5)	C3—C2—C1	119.6 (2)
O2^iii^—Zn1—O1	95.17 (6)	C4—C2—C1	121.69 (19)
O2^ii^—Zn1—O1	90.75 (6)	C2^v^—C3—C2	120.5 (3)
N1^iv^—Zn1—O1	84.42 (4)	C2^v^—C3—H3	119.7
O2^iii^—Zn1—O1^i^	90.75 (6)	C2—C3—H3	119.7
O2^ii^—Zn1—O1^i^	95.17 (6)	N1—C4—C2	120.30 (19)
N1^iv^—Zn1—O1^i^	84.42 (4)	N1—C4—C5	117.2 (2)
O1—Zn1—O1^i^	168.83 (9)	C2—C4—C5	122.51 (19)
C4—N1—C4^v^	121.2 (3)	C4—C5—H5A	109.5
C4—N1—Zn1^iv^	119.41 (13)	C4—C5—H5B	109.5
C4^v^—N1—Zn1^iv^	119.41 (13)	H5A—C5—H5B	109.5
C1—O1—Zn1	124.94 (16)	C4—C5—H5C	109.5
C1—O2—Zn1^iii^	117.23 (15)	H5A—C5—H5C	109.5
O1—C1—O2	125.3 (2)	H5B—C5—H5C	109.5
O1—C1—C2	118.7 (2)		
			
O2^iii^—Zn1—O1—C1	120.17 (19)	O2—C1—C2—C4	−137.8 (2)
O2^ii^—Zn1—O1—C1	4.03 (18)	C4—C2—C3—C2^v^	−3.21 (13)
N1^iv^—Zn1—O1—C1	−118.08 (18)	C1—C2—C3—C2^v^	173.1 (2)
O1^i^—Zn1—O1—C1	−118.08 (18)	C4^v^—N1—C4—C2	−3.33 (14)
Zn1—O1—C1—O2	−103.1 (2)	Zn1^iv^—N1—C4—C2	176.67 (14)
Zn1—O1—C1—C2	74.7 (2)	C4^v^—N1—C4—C5	175.2 (2)
Zn1^iii^—O2—C1—O1	−0.8 (3)	Zn1^iv^—N1—C4—C5	−4.8 (2)
Zn1^iii^—O2—C1—C2	−178.63 (14)	C3—C2—C4—N1	6.6 (3)
O1—C1—C2—C3	−131.9 (2)	C1—C2—C4—N1	−169.61 (17)
O2—C1—C2—C3	46.1 (3)	C3—C2—C4—C5	−171.85 (17)
O1—C1—C2—C4	44.2 (3)	C1—C2—C4—C5	11.9 (3)

Symmetry codes: (i) −*x*, *y*, −*z*+1/2; (ii) *x*, −*y*, *z*−1/2; (iii) −*x*, −*y*, −*z*+1; (iv) −*x*+1/2, −*y*+1/2, −*z*+1; (v) −*x*+1, *y*, −*z*+3/2.

### Hydrogen-bond geometry (Å, °)

**Table:**

Cg is the centroid of the pyridine ring.

**Table:**

*D*—H···*A*	*D*—H	H···*A*	*D*···*A*	*D*—H···*A*
C5—H5C···Cg^iv^	0.96	2.67	3.573 (4)	158.

Symmetry codes: (iv) −*x*+1/2, −*y*+1/2, −*z*+1.
